# Assessing Renal Function in Chronic Kidney Disease: A Comparative Evaluation of Glomerular Filtration Rate Prediction Equations in the North-Central Region of Nigeria

**DOI:** 10.7759/cureus.84577

**Published:** 2025-05-21

**Authors:** Olawale Bakare, Emmanuel I Agaba, Zumnan M Gimba, Esala E Abene, Lucius Imoh, Joseph Maji

**Affiliations:** 1 Renal Medicine, New Cross Hospital, Wolverhampton, GBR; 2 Medicine, Jos University Teaching Hospital, Jos, NGA; 3 Internal Medicine, Jos University Teaching Hospital, Jos, NGA; 4 Chemical Pathology, University of Jos, Jos, NGA; 5 Nephrology, Federal Medical Centre, Keffi, NGA; 6 Internal Medicine/Nephrology, Jos University Teaching Hospital, Jos, NGA

**Keywords:** albuminuria, chronic kidney disease epidemiology collaboration (ckd-epi), clinical epidemiology, cockroft-gault (cg), creatinine clearance, gfr estimation, kidney disease management, modification of diet in renal disease (mdrd), nigerian ckd patients, renal function assessment

## Abstract

Background: The glomerular filtration rate (GFR) is considered the gold standard for assessing renal function. According to the Kidney Disease: Improving Global Outcomes (KDIGO) guidelines, GFR estimation is often carried out using predictive equations that incorporate serum creatinine levels, along with demographic factors such as age, gender, race, and body size. However, these equations exhibit varying levels of accuracy across different populations, necessitating the evaluation of their performance and clinical relevance in diverse patient groups.

Objectives: This study aimed to evaluate the performance of three commonly used GFR estimation equations, the Cockcroft-Gault (CG), Modification of Diet in Renal Disease (MDRD), and Chronic Kidney Disease Epidemiology Collaboration (CKD-EPI) equations, along with their race-modified versions, by comparing them with measured 24-hour creatinine clearance (CrCl) in adult patients with chronic kidney disease (CKD).

Methods: A cross-sectional descriptive study was conducted at Jos University Teaching Hospital (JUTH) between November 2019 and July 2020, involving 111 consecutively recruited CKD patients. Data collection included medical history, physical examination, laboratory investigations, calculation of CrCl, estimated glomerular filtration rate (eGFR) using different equations, and albumin-creatinine ratio (ACR). Statistical analysis was performed using SPSS version 20 (IBM Inc., Armonk, New York), with p-values <0.05 considered statistically significant.

Results: The mean age of participants was 51.1±15.5 years. Diabetes mellitus was the most common cause of CKD, affecting 38% of the cohort. Among participants, 31% and 22% were classified as having stage 5 and stage 4 CKD, respectively. The median CrCl was 26 (9-56) mL/min. The median eGFR values across the equations were as follows: CG, 26 (11-60) mL/min/1.73m²; MDRD, 26 (11-60) mL/min/1.73m²; MDRD1, 22 (9-50) mL/min/1.73m²; CKD-EPI, 26 (10-62) mL/min/1.73m²; and CKD-EPI1, 22 (9-53) mL/min/1.73m². Strong positive correlations were observed between measured CrCl and the estimated GFR from each equation: CG (r=0.948, p=0.001), MDRD (r=0.940, p=0.001), MDRD1 (r=0.939, p=0.001), CKD-EPI (r=0.943, p=0.001), and CKD-EPI1 (r=0.942, p=0.001). Furthermore, significant correlations were found between the different GFR equations themselves, with the highest correlation observed between MDRD and CKD-EPI (r=0.999, p=0.001). The median ACR was 395.5 (45.3-2887.0) mg/g, and albuminuria was present in 82% of participants. All three GFR equations closely approximated the measured CrCl of 26 mL/min/1.73m². The equations performed optimally in patients with GFR values below 45 mL/min, with the CG equation exhibiting the least bias and the highest precision. Regression analysis revealed a significant association between albuminuria and all GFR equations and a notable association between age and CrCl, CG, and CKD-EPI estimates.

Conclusion: The CG, MDRD, and CKD-EPI equations demonstrated comparable accuracy to measured 24-hour CrCl in estimating GFR in Nigerian patients with CKD. These findings support the clinical use of these predictive equations for renal function assessment in CKD, though local validation in diverse populations is recommended for optimal clinical application.

## Introduction

Chronic kidney disease (CKD) is a clinical syndrome marked by abnormalities in kidney structure or function persisting for over three months, with significant implications for overall health [1,2]. According to the National Kidney Foundation's Kidney Disease Outcomes Quality Initiative (NKF/KDOQI), CKD is diagnosed when kidney damage lasts more than three months, with or without reduced glomerular filtration rate (GFR). Diagnosis is established through pathological abnormalities, markers of kidney damage, or a history of kidney transplantation. Alternatively, CKD is identified when an individual's GFR remains below 60 mL/min/1.73 m² for three months or longer, even in the absence of overt kidney damage [3].

The global burden of CKD is escalating, with an annual increase of 8%, disproportionately affecting developing nations [4,5]. In 2015, the World Health Organization (WHO) estimated 1.2 million deaths from renal failure globally, with the current toll reaching 5-10 million deaths annually [6]. In Africa, the CKD prevalence is estimated at 15.8%, with sub-Saharan Africa bearing the heaviest burden [7]. Nigeria, in particular, faces a high CKD prevalence, with rates ranging from 1.6% to 12.4% across various studies [8].

Estimating GFR from serum creatinine levels is a standard practice, using equations that account for age, sex, race, and body size to reflect creatinine generation [9,10]. The Cockroft-Gault (CG) formula, introduced in 1976, estimates creatinine clearance (CrCl) based on these factors but has limitations, including imprecision at higher GFR levels [11,12]. It estimates CrCl rather than true GFR, leading to an overestimation due to unaccounted creatinine secretion [12,13]. Additionally, the CG formula was developed using older creatinine assay methods, which may produce inaccuracies with modern techniques [11,13].

The Modification of Diet in Renal Disease (MDRD) study equation, developed from a multi-center trial involving 1,628 CKD patients, estimates GFR using serum creatinine, age, sex, and race [13]. While validated in African-Americans, diabetic nephropathy patients, and transplant recipients, the MDRD equation tends to underestimate GFR in individuals with higher renal function, potentially leading to CKD over-diagnosis [14]. To address these limitations, the Chronic Kidney Disease Epidemiology Collaboration (CKD-EPI) equation was developed in 2009 [13]. Using the same four variables, the CKD-EPI equation provides more accurate GFR estimates, particularly for values above 60 mL/min/1.73 m², and is now recommended by the National Kidney Foundation and Kidney Disease: Improving Global Outcomes (KDIGO) guidelines for adult GFR estimation [15].

The accuracy of these predictive equations depends on their correlation with measured GFR, but concerns arise regarding their applicability across diverse populations. Most equations were developed in Caucasian and African-American populations with a racial correction factor, whose validity for other populations of African ancestry is debated [16]. Studies in healthy Congolese adults, Malawian HIV-positive and HIV-negative individuals, and Ghanaian cohorts suggest that omitting the racial correction factor may improve equation performance [17-20]. Similarly, Nigerian studies indicate that the CG and MDRD equations reliably estimate GFR in CKD patients, with stronger correlations between the MDRD and CKD-EPI equations [21]. However, research in sickle cell disease patients has shown correlations between the CKD-EPI and CG formulas [22], emphasizing the need for validation in various clinical populations.

Given the KDIGO guidelines' endorsement of equations like CG, MDRD, and CKD-EPI for GFR estimation, it is crucial to assess their performance in Nigerian CKD patients. Ensuring accurate diagnosis and staging through validated predictive equations is essential for optimizing patient care and health outcomes in this high-burden population.

Aim

This study aimed to evaluate the performance of the three commonly used GFR estimation equations - Cockcroft-Gault (CG), MDRD, and CKD-EPI - with and without race modifications, compared to the 24-hour CrCl measurement in adult patients with CKD.

## Materials and methods

Between November 2019 and July 2020, a hospital-based cross-sectional study was conducted at Jos University Teaching Hospital (JUTH), a 520-bed facility offering both inpatient and outpatient services. Eligible and consenting patients with CKD aged 18 years and older who were accessing nephrology services at JUTH were recruited consecutively through a convenience sampling method. The sample size was calculated using Modified Cochran’s formula, yielding a minimum of 102 participants, though 111 patients were ultimately recruited to enhance the robustness of the study.

Participants were eligible for inclusion if they were 18 years or older and had stable renal function, defined as no significant change in estimated glomerular filtration rate (eGFR) or serum creatinine levels over the preceding four weeks. Patients with acute kidney injury (AKI), those undergoing dialysis for end-stage renal disease (ESRD), individuals with congestive cardiac failure, pregnant women, and renal transplant recipients were excluded to ensure a well-defined study population for accurate assessment of kidney function and the applicability of predictive equations in CKD diagnosis and staging.

Data collection involved the use of consent forms and structured questionnaires to capture relevant clinical and demographic information (Appendices A and B (Table 11)). For sample collection and physical assessments, various materials and equipment were utilized, including 5 L wide-mouth containers for urine collection, measuring jars, plain sample bottles for serum and urine creatinine determination, sterile needles and syringes, tourniquets, latex gloves, cotton wool, methylated spirit, and plaster. Weight and height measurements were taken using a SECA bathroom scale and SECA stadiometer (SECA GmbH & Co. KG, Hamburg, Germany), while blood pressure was measured using an Accoson sphygmomanometer (Accoson Ltd., Essex, UK) with appropriately sized cuffs.

Participants were interviewed by the researcher to collect demographic data, medical history, and duration of illness. Information on etiological diagnosis, family history of CKD, comorbidities, and drug history was documented, with any recall difficulties addressed by extracting relevant data from the participant's medical records. Consenting participants were admitted to the ward on a predetermined date for 24-hour urine collection and blood sample collection. They were instructed to void at zero hours, discarding the initial urine sample, while subsequent samples were collected in wide-mouth containers containing boric acid until the 24th hour when the final sample was obtained. The entire collection process was closely supervised to ensure accuracy and completeness. After urine collection, approximately 5 mL of venous blood was drawn from each participant into plain bottles, after which they were discharged home.

The total volume of urine collected over 24 hours in the laboratory was measured using a calibrated measuring jar. Two-milliliter aliquots of urine were taken and stored in plain bottles for further analysis. Blood samples were centrifuged to separate serum, and serum and urinary creatinine were analyzed using a kinetic colorimetric assay based on Jaffe's method with an automated Cobas C111 chemistry analyzer (Roche Diagnostics, Mannheim, Germany). This assay involves the formation of a yellow-red complex between creatinine and picrate in an alkaline solution, with the rate of dye formation proportional to the creatinine concentration in the specimen. The method incorporates "rate-blanking" to minimize interference from bilirubin and corrects for non-specific reactions caused by pseudo-creatinine chromogens, such as proteins and ketones.

Urinary albumin was measured using an immunofluorescence method on a semi-automated Ichroma Analyzer (Boditech Med. Inc, South Korea). This assay utilizes a sandwich immunodetection method, where a labeled detector antibody binds to albumin in the urine sample, forming an antigen-antibody complex. This complex is subsequently captured by another immobilized labeled antibody for detection, with the intensity of fluorescence directly proportional to the albumin concentration in the urine sample.

This meticulous methodology, from patient recruitment to sample analysis, ensured reliable data collection and accurate laboratory assessments, providing a robust framework for evaluating kidney function and the role of predictive equations in CKD diagnosis and staging. Operational and statistical definitions are in Appendix C. 

Determination of GFR using 24-hour CrCl

The GFR was calculated using the following formula:

$CrCl = UCr * V/PCr$

(CrCl = creatinine clearance (mL/min), UCr = urinary concentration of creatinine (mg/dL), V = urine flow rate (volume voided per minute, 1440 minutes for 24-hour collection), PCr = serum creatinine concentration (mg/dL)).

To standardize the measured CrCl for each participant, the value was corrected to a body surface area (BSA) of 1.73 m² using the following equation:

$Corrected CrCl = CrCl (Measured 24-hour)* BSA/1.73m2$

CrCl was used as the reference standard for this study. 

Determination of eGFR using the CG equation

eGFR was calculated using the CG equation as follows:

$eGFR = (140 - Age * weight)/ 0.814 * Scr(umol/L)$ for men,and;

 $(140 - Age * weight)/ 0.814 * Scr(umol/L) * 0.85$ in women

(Age = age of the participant in years, Weight = weight of the participant in kg, Scr = serum creatinine concentration in µmol/L).

The electronic version of the CG equation was used for all participants. The predicted estimates for each participant were corrected to a BSA of 1.73 m² for uniformity with other equations and the measured 24-hour CrCl as follows:

$CG= estimated CG * BSA/1.73m2$

Determination of EGFR using the MDRD and MDRD1 equations

The eGFR was determined using the MDRD equations. The electronic calculator app was employed with inputs of serum creatinine, age, sex, and race. The manual calculation is outlined below:

$GFR(mL/min/1.73m2) = 30,849 * SCr(umol/L)-1.154 * age-0.203 * 0.742(if female) * 1.210 (if black)$

$GFR(mL/min/1.73m2) = 30,849 * SCr(umol/L)-1.154 * age-0.203 * 0.742(if female)$

Two results were obtained by including race in the equation (MDRD (Black)) and without race adjustment (MDRD1).

Determination of eGFR using the CKD-EPI and CKD-EPI1 equations

The eGFR was determined using the CKD-EP collaboration equations. The electronic calculator app was used with inputs of serum creatinine, age, sex, and race. The manual calculation formula is as follows:

$GFR (mL/min/1.73m2) = 141 * min (SCr/k, 1) * max(SCr/k, 1)1.209 * 0.933age * 1.018(if female) * 1.57(if black)$

The formula without race correction is $GFR (mL/min/1.73m2) = 141 * min (SCr/k, 1) * max(SCr/k, 1)1.209 * 0.933age * 1.018(if female)$

Results were generated with and without race input, producing the CKD-EPI and chronic kidney disease epidemiology collaboration without race correction (CKD-EPI1) estimates, respectively.

ACR

Two urine aliquots were collected to measure albumin concentration (mg) and creatinine concentration (g). The albumin-to-creatinine ratio (ACR) was expressed in mg/g, reflecting the daily protein excretion in milligrams.

Ethical considerations

Ethical clearance for the study was obtained from the Human Research and Ethical Committee of JUTH with approval number JUTH/DCS/ADM/127/XXVII/923 prior to the commencement of data collection. Informed consent was obtained from all participants before their inclusion in the study, and confidentiality was strictly maintained throughout the research process. Participants were fully informed of their right to withdraw or decline participation at any stage, with the assurance that their decision would be respected and would not affect the quality or continuity of their medical care. Furthermore, all investigations and hospital admissions related to the study were provided at no cost to the participants, ensuring equitable access to the necessary assessments without financial burden.

Data and statistical analysis

The data obtained from the questionnaires were meticulously edited, cleaned, coded, and entered into Microsoft Excel for initial processing. Subsequent data analysis was conducted using the Statistical Package for the Social Sciences (SPSS), version 20 (Chicago, Illinois, USA). Quantitative variables, such as age, weight, height, body mass index (BMI), and BSA, were summarized using means and standard deviations (SDs), while non-normally distributed variables, including the duration of CKD, urine volume, and other skewed measurements, were expressed as medians with interquartile ranges (IQRs). Categorical variables, such as sex and place of residence, were presented as percentages to provide a clear demographic overview of the study population.

To compare the mean eGFR derived from each equation across different GFR stages, analysis of variance (ANOVA) was employed. The Wilcoxon signed-rank test was used to compare the median values of each equation with the measured 24-hour CrCl, offering insight into how closely each equation approximated actual renal function. Spearman's rank correlation was applied to assess associations between 24-hour CrCl, urinary ACR, and the various eGFR equations, allowing for an evaluation of the strength and direction of these relationships.

Bland-Altman plots were generated to visually and statistically evaluate the performance of the equations against measured 24-hour CrCl, facilitating the determination of bias, precision, and accuracy. Agreement between the equations was further assessed using Spearman’s rank correlation, ensuring a robust comparison of their clinical applicability. Regression analysis was performed to investigate the correlation between eGFR equations and socio-demographic as well as cardiovascular risk factors, including age, sex, place of residence, hypertension, diabetes, and the presence of albuminuria. A p-value of less than 0.05 was considered statistically significant, with the confidence interval set at 95%. This rigorous statistical approach ensured a comprehensive evaluation of the equations’ validity and their potential utility in CKD diagnosis and management.

## Results

Socio-demographic characteristics of study participants

The mean±standard deviation (SD) age of the participants was 51.1±15.5 years. More than half, 58 (52.3%) of the participants were male. Among the 111 participants, 67 (60.4%) were employed, while the remaining 44 (39.6%) were either unemployed or retired. The majority of the participants, 107 (96.4%), resided in urban areas, while four (3.6%) lived in rural settings.

Clinical characteristics of study participants

The median duration of CKD among the participants was 12.6 months, with an IQR of six to 48 months. A majority of the participants, 93 (83.8%), reported no known family history of CKD, while 18 (16.2%) had at least one family member affected by the condition. The etiological factors for CKD are presented in Table 1. The study found that 92 patients (82.9%) had hypertension as the most common co-morbidity. Additionally, one (0.9%) participant with hypertensive nephrosclerosis also had diabetes mellitus. Other comorbidities included chronic liver disease in two (1.8%) participants, obstructive uropathy in six (5.4%), connective tissue disease in one (0.9%), and retroviral disease in one (0.9%). Regarding medications, the most commonly used were angiotensin-converting enzyme (ACE) inhibitors or angiotensin receptor blockers (ARBs) (95 patients (85.6%)), diuretics (86 patients (77.5%)), and calcium channel blockers (75 patients (67.6%)).

**Table 1:** Socio-demographic characteristics and causes of CKD DM = diabetes mellitus, CGN = chronic glomerulonephritis, HTN = hypertension, ADPKD = autosomal polycystic kidney disease, MCD = minimal change disease, IGA = immunoglobulin A, SLE = systemic lupus erythematosus

Variable	Frequency	Percent
Mean age (51.1±15.5 years)		
Sex		
Male	58	52.3
Female	53	47.7
Employment status		
Employed	67	60.4
Unemployed or retired	44	39.6
Settlement pattern		
Urban	107	96.4
Rural	4	3.6
Median duration of CKD: 12.6 months (IQR: 6-48 months)		
Family history of CKD		
No known family history	93	83.8
At least one family member affected by CKD	18	16.2
Causes of CKD		
DM	42	37.8
CGN	26	23.4
HTN	14	12.6
ADPKD	11	9.9
Obstructive nephropathy	9	8.1
Unknown	3	2.7
MCD	2	1.8
Multiple myeloma	1	0.9
IGA nephropathy	1	0.9
Sickle cell nephropathy	1	0.9
SLE	1	0.9

Physical examination

The overall mean±SD weight of the participants was 68.6±13.0 kg. Male participants had a mean weight of 69.5±10.9 kg, while female participants had a mean weight of 67.7±15.1 kg. This difference was not statistically significant (p=0.464). The overall mean height was 1.64±0.09 m, with males having significantly greater mean height than females (p=0.017). In contrast, female participants had a significantly higher mean BMI of 27.4±6.1 kg/m² compared to male participants (p=0.001) (Table 2). The overall mean BSA was 1.7±0.3 m², with no statistically significant difference between male and female participants (p=0.543). The overall mean systolic blood pressure (SBP) was 148.5±34.1 mmHg, while the overall mean diastolic blood pressure (DBP) was 91.2±20.2 mmHg (Table 2).

**Table 2:** Anthropometric and clinical characteristics *Significant SD = standard deviation, IQR = interquartile range, T = student t test, U = Mann-Whitney U, BMI = body mass index, BSA = body surface area, SBP = systolic blood pressure, DBP = diastolic blood pressure

Variables	Overall	Male	Female	T-test	p-value
Weight (kg), mean±SD	68.6±13.0	69.5±10.9	67.7±15.1	0.735	0.464
Height (m), mean±SD	1.64±0.09	1.70±0.06	1.58±0.06	10.848	<0.001*
BMI (kg/m^2^), mean±SD	25.7±5.3	24.1±3.9	27.4±6.1	3.358	0.001*
BSA (m^2^), mean±SD	1.7±0.3	1.8±0.3	1.7±0.2	0.611	0.543
SBP (mm/Hg), mean±SD	148.5±34.1	151.6±27.7	145.0±40.0	1.013	0.313
DBP (mm/Hg), mean±SD	91.2±20.2	93.9±21.1	88.1±18.9	1.547	0.125
Duration of CKD (months), median (IQR)	12.4 (6-48)	12 (6-36)	12 (6.5-66.0)	U=1395.000	0.362

Laboratory variables of study participants

Dipstick urinalysis revealed that 84 participants (75.7%) had proteinuria. The overall median urine volume over 24 hours was 2020 mL, with an IQR of 1320-2920 mL. The median urine albumin concentration was 168.0 mg/L, with an IQR of 27.0-1758.3 mg/L. The median serum creatinine level was 219.0 µmol/L, with an IQR of 108.0-536.0 µmol/L. The median urinary creatinine concentration was 4220.0 µmol/L, with an IQR of 3240.0-6390.0 µmol/L. The median ACR was 395.5 mg/g, with an IQR of 45.3-2887.0 mg/g (Table 3).

**Table 3:** Laboratory findings IQR = interquartile range, Cr = creatinine, ACR = albumin creatinine ratio

Urinalysis	N (%) OR median (IQR)
Dipstix protein	
Yes	84 (75.7)
No	27 (24.3)
Urine volume in 24 hours (mL)	2020.0 (1320.0-2920.0)
Urine albumin concentration (mg/L)	168.0 (27.0-1758.3)
Urine Cr (umol/L)	4220.0 (3240.0-6390.0)
ACR (mg/g)	395.5 (45.3-2887.0)
Serum Cr (umol/L)	219.0 (108.0-536)

CrCl and eGFR calculated from the various equations

The median CrCl of the participants was 26 mL/min/1.73 m², with an IQR of 9-56 mL/min. The median values for the eGFR calculated using the various equations are presented in Table 4.

**Table 4:** CrCl and the eGFR from various equations (n=111) *Significant Ref. = reference or baseline group, CG = Cockroft-Gault, MDRD = Modification of Diet in Renal Disease, MDRD1 = Modification of Diet in Renal Disease without race correction, CKD-EPI = Chronic Kidney Disease Epidemiology Collaboration, CKD-EPI1 = Chronic Kidney Disease Epidemiology Collaboration without race correction, IQR = interquartile range, W-test = Wilcoxon signed-rank test, CrCl = creatinine clearance

Equation	Median (IQR)	W-test	P-value
CrCl	26 (9-56)	Ref.	Ref.
CG	26 (12-54)	1.261	0.207
MDRD	26 (11-60)	1.672	0.095
MDRD1	22 (9-50)	3.924	<0.001*
CKD-EPI	26 (10-62)	1.770	0.077
CKD-EPI1	22 (9-53)	2.332	0.020*

CKD stages

**Figure 1:**
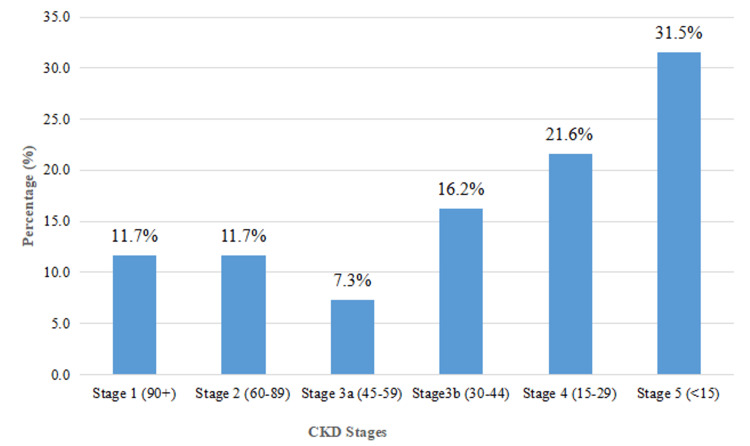
CKD stages with percentage distribution CKD, chronic kidney disease

Based on the median CrCl, participants were classified into five stages of CKD. The majority were in stage 5 CKD (35 participants (31.5%)), followed by stage 4 (24 (21.6%)), stage 3b (18 (16.2%)), stages 1 and 2 (13 each (11.7%)), and stage 3a (8 (7.3%)) (Figure 1).

Relationship between CrCl and the various equations

**Figure 2:**
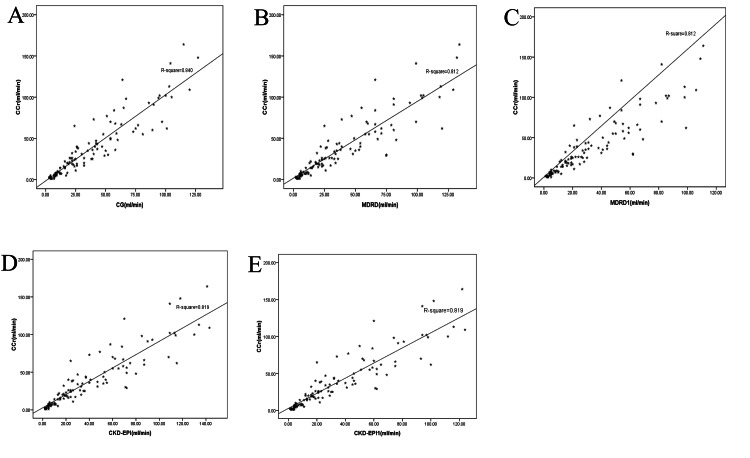
Plot of measured 24-hour CrCl against the various equations A = plot of measured 24-hour CrCl against CG-predicted GFR values; B = plot of measured 24-hour CrCl against MDRD-predicted GFR values; C = plot of measured 24-hour CrCl against MDRD1-predicted GFR values; D = plot of measured 24-hour CrCl against CKD-EPI-predicted GFR values; E = plot of measured 24-hour CrCl against CKD-EPI1-predicted GFR values CrCl = creatinine clearance, CG = Cockroft-Gault, GFR = glomerular filtration rate, MDRD = Modification of Diet in Renal Disease, MDRD1 = Modification of Diet in Renal Disease without race correction, CKD-EPI = Chronic Kidney Disease Epidemiology Collaboration, CKD-EPI1 = Chronic Kidney Disease Epidemiology Collaboration without race correction

The study demonstrated a strong positive correlation between CrCl and the various eGFR equations. CrCl showed a robust positive correlation with the CG equation (Spearman’s correlation, r=0.948, p=0.001), the MDRD equation (r=0.940, p=0.001), MDRD1 (r=0.939, p=0.001), CKD-EPI (r=0.943, p=0.001), and CKD-EPI1 (r=0.942, p=0.001). Linear regression plots of the various equations against 24-hour CrCl indicated a significant positive correlation for all equations: CG (r=0.917), MDRD (r=0.901), MDRD1 (r=0.901), CKD-EPI (r=0.905), and CKD-EPI1 (r=0.905) (Figure 2).

**Figure 3:**
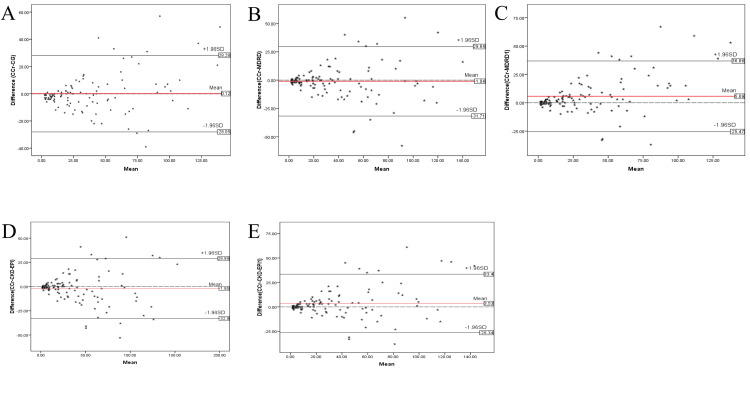
The mean difference between 24-hour CrCl against the various equations A = plot showing the difference between the CG-predicted CrCl and the measured 24-hour CrCl. B = plot showing the difference between the MDRD-predicted GFR and the measured 24-hour CrCl; C = plot showing the difference between the MDRD1-predicted GFR and the measured 24-hour CrCl. D = plot showing the difference between the CKD-EPI-predicted GFR and the measured 24-hour CrCl; E = plot showing the difference between the CKD-EPI1-predicted GFR and the measured 24-hour CrCl CrCl = creatinine clearance, CG = Cockroft-Gault, GFR = glomerular filtration rate, MDRD = Modification of Diet in Renal Disease, MDRD1 = Modification of Diet in Renal Disease without race correction, CKD-EPI = Chronic Kidney Disease Epidemiology Collaboration, CKD-EPI1 = Chronic Kidney Disease Epidemiology Collaboration without race correction

The mean difference between the CG-predicted CrCl and the measured 24-hour CrCl was 0.12±14.40 mL/min, with discordance observed at CrCl values >48 mL/min. Eight measurements were outside the 95% confidence interval. The mean difference between the MDRD-predicted GFR and the measured 24-hour CrCl was -1.04±15.65 mL/min, with discordance at CrCl values >45 mL/min. Nine measurements were outside the 95% confidence interval. The mean difference between the MDRD1-predicted GFR and the measured 24-hour CrCl was 5.69±15.90 mL/min, with discordance at CrCl values >45 mL/min. Ten measurements were outside the 95% confidence interval. The mean difference between the CKD-EPI-predicted GFR and the measured 24-hour CrCl was -1.95±15.80 mL/min, with discordance at CrCl values >48 mL/min. Ten measurements were outside the 95% confidence interval. The mean difference between the CKD-EPI1-predicted GFR and the measured 24-hour CrCl was -3.53±15.24 mL/min, with discordance at CrCl values >45 mL/min. Eleven measurements were outside the 95% confidence interval (Figure 3).

The findings in Table 5 show the number of measurements within the 30% limits of the measured CrCl. MDRD1 exhibited the highest accuracy, followed by CKD-EPI1. CG and MDRD showed similar accuracy, while CKD-EPI demonstrated the lowest accuracy.

**Table 5:** Measurement of the performance of the various equations: CG, MDRD, MDRD1, CKD-EPI, and CKD-EPI1 compared to 24-hour CrCl CrCl = creatinine clearance, CG = Cockroft-Gault, MDRD = Modification of Diet in Renal Disease, MDRD1 = Modification of Diet in Renal Disease without race correction, CKD-EPI = Chronic Kidney Disease Epidemiology Collaboration, CKD-EPI1 = Chronic Kidney Disease Epidemiology Collaboration without race correction

Equation	Bias (mL/min/1.73m^2^)	Precision	Accuracy (%)
CG	0.12	14.4	59.5
MDRD	-1.04	15.65	59.5
MDRD1	5.69	15.90	62.2
CKD-EPI	-1.95	15.80	58.6
CKD-EPI1	3.53	15.24	61.3

Relationship between urinary ACR and the various equations

Urinary ACR showed a negative correlation with all the eGFR equations. Specifically, the correlations were as follows: CG (r=-0.520, p=0.001), MDRD (r=-0.528, p=0.001), MDRD1 (r=-0.527, p=0.001), CKD-EPI (r=-0.527, p=0.001), and CKD-EPI1 (r=-0.525, p=0.001) (Table 6).

**Table 6:** Relationship between CrCl, urinary ACR, and the various equations (CG, MDRD, MDRD1, CKD-EPI, and CKD-EPI1) *Significant CrCl = creatinine clearance, CG = Cockroft-Gault, MDRD = Modification of Diet in Renal Disease, MDRD1 = Modification of Diet in Renal Disease without race correction, CKD-EPI = Chronic Kidney Disease Epidemiology Collaboration, CKD-EPI1 = Chronic Kidney Disease Epidemiology Collaboration without race correction

Equations	CrCl		Urinary ACR	
	Rho	P-value	Rho	P-value
CG	0.948	<0.001*	-0.520	<0.001*
MDRD	0.940	<0.001*	-0.528	<0.001*
MDRD1	0.939	<0.001*	-0.527	<0.001*
CKD-EPI	0.943	<0.001*	-0.527	<0.001*
CKD-EPI1	0.942	<0.001*	-0.525	<0.001*

Relationship between the various equations (CG, MDRD, MDRD1, CKD-EPI, and CKD-EPI1) at different CKD stages

A positive correlation was observed between the measured 24-hour CrCl and CG-predicted CrCl at stage 1 of the GFR classification (Spearman's correlation, r=0.602, p=0.029) (Table 7). However, no statistically significant correlation was found between the measured 24-hour CrCl and the predicted GFR from the other equations at stages 1-3b. Additionally, CG did not correlate with the measured 24-hour CrCl estimate of GFR from stages 2-4.

**Table 7:** Relationship between CrCl and various equations (CG, MDRD, MDRD1, CKD-EPI, and CKD-EPI1) at different GFR stages *Significant CCrCl = creatinine clearance, CG = Cockroft-Gault, GFR = glomerular filtration rate, MDRD = Modification of Diet in Renal Disease, MDRD1 = Modification of Diet in Renal Disease without race correction, CKD-EPI = Chronic Kidney Disease Epidemiology Collaboration, CKD-EPI1 = Chronic Kidney Disease Epidemiology Collaboration without race correction

Equations	Stage 1		Stage 2		Stage 3a		Stage 3b		Stage 4		Stage 5	
	Rho	P-value	Rho	P-value	Rho	P-value	Rho	P-value	Rho	P-value	Rho	P-value
CG	0.602	0.029*	-0.441	0.132	0.518	0.188	0.076	0.765	0.076	0.765	0.815	<0.001*
MDRD	0.556	0.048	-0.499	0.082	0.204	0.629	0.005	0.984	0.005	0.984	0.825	<0.001*
MDRD1	0.546	0.053	-0.515	0.072	0.204	0.629	0.012	0.961	0.012	0.961	0.804	<0.001*
CKD-EPI	0.445	0.128	-0.505	0.782	0.157	0.711	-0.024	0.925	-0.024	0.925	0.828	<0.001*
CKD-EPI1	0.445	0.128	-0.505	0.782	0.157	0.711	-0.032	0.901	-0.032	0.901	0.823	<0.001*

All prediction equations demonstrated a significant correlation with the measured 24-hour CrCl estimate of GFR at stages 4 and 5. Among these, CKD-EPI showed the best correlation at stage 5 (r=0.828, p<0.001) (Table 7). Notably, no correlation was observed between the measured 24-hour CrCl and CG at the stage 4 GFR category.

The means±SD for the measured 24-hour CrCl, predicted CrCl by the CG equation, and estimated GFRs by MDRD, MDRD1, CKD-EPI, and CKD-EPI1 at stage 1 of the GFR classification were as follows: measured 24-hour CrCl: 117.4±25.5 mL/min, CG-predicted CrCl: 99.5±21.9 mL/min, MDRD: 100.1±24.7 mL/min, MDRD1: 82.8±20.4 mL/min, CKD-EPI: 105.7±25.6 mL/min, and CKD-EPI1: 91.2±22.3 mL/min. The analysis of variance (ANOVA) yielded a significant difference between the groups (F=3.339, p=0.009).

There was no significant difference between the mean GFR estimates from the measured 24-hour CrCl and the various equations (CG, MDRD, MDRD1, CKD-EPI, and CKD-EPI1) at stages 2, 3a, 3b, 4, and 5. The results of ANOVA for these stages were as follows: stage 2: F=0.955 (p=0.450), stage 3a: F=0.364 (p=0.869), stage 3b: F=1.063 (p=0.385), stage 4: F=0.939 (p=0.458), and stage 5: F=2.229 (p=0.053) (Table 8).

**Table 8:** Relationship between CrCl and various equations (CG, MDRD, MDRD1, CKD-EPI, and CKD-EPI1) at different GFR stages *Significant CCrCl = creatinine clearance, CG = Cockroft-Gault, GFR = glomerular filtration rate, MDRD = Modification of Diet in Renal Disease, MDRD1 = Modification of Diet in Renal Disease without race correction, CKD-EPI = Chronic Kidney Disease Epidemiology Collaboration, CKD-EPI1 = Chronic Kidney Disease Epidemiology Collaboration without race correction, F (ANOVA) = analysis of variance

CKD stages (mean±SD)	Creatine clearance (mean±SD)	CG (mean±SD)	MDRD (mean±SD)	MDRD1 (mean±SD)	CKD-EPI (mean±SD)	CKD-EPI1 (mean±SD)	F-test (P-value)
Stage 1	117.4±25.5	99.5±21.9	100.1±24.7	82.8±20.4	105.7±25.6	91.2±22.3	3.339(0.009)*
Stage 2	71.0±8.9	71.3±23.7	70.5±26.5	58.3±21.8	74.1±28.7	64.0±25.0	0.955(0.450)
Stage 3a	51.7±4.8	50.8±9.3	54.3±20.7	45.2±17.2	55.3±19.4	47.7±16.7	0.364(0.869)
Stagg 3b	38.5±12.8	40.4±11.8	41.3±15.3	34.1±12.7	41.4±15.6	35.6±13.3	1.063(0.385)
Stage 4	23.1±10.5	24.6±8.8	25.5±12.9	21.0±10.8	24.7±12.6	21.4±11.0	0.939(0.458)
Stage 5	6.0±4.7	9.1±5.1	7.5±5.1	6.3±4.3	7.2±5.0	6.2±4.4	2.229(0.053)
Overall mean	39.0±37.1	38.9±32.9	38.9±34.6	32.2±28.6	39.9±36.6	34.4±31.6	0.963(0.440)
F (ANOVA)	292.241	109.477	76.990	77.145	81.842	81.182	-
P-value	<0.001*	<0.001*	<0.001*	<0.001*	<0.001*	<0.001*	-

Correlation and agreement between the various equations

The relationship between the various equations, CG, MDRD, MDRD1, CKD-EPI, and CKD-EPI1, was assessed using Spearman's correlation, as detailed in Table 9. The results demonstrated generally strong positive correlations between the equations, indicating a high degree of agreement. This suggests that all the equations provide similar estimates of kidney function, with little variation in the results when comparing the different methods.

**Table 9:** Correlation showing agreement between the various equations; CG, MDRD, MDRD1, CKD-EPI, and CKD-EPI1 *Significant Ref. = Reference or baseline group, CG = Cockroft-Gault, GFR = glomerular filtration rate, MDRD= Modification of Diet in Renal Disease, MDRD1 = Modification of Diet in Renal Disease without race correction, CKD-EPI = Chronic Kidney Disease Epidemiology Collaboration, CKD-EPI1 = Chronic Kidney Disease Epidemiology Collaboration without race correction, rho = Spearman’s correlation coefficient

Equation	Coef.	Equations				
		CG	MDRD	MDRD1	CKD-EPI	CKD-EPI1
CG	Rho	Ref.	0. 985	0. 984	0. 989	0. 988
	p-value	Ref.	<0.001*	<0.001*	<0.001*	<0.001*
MDRD	Rho	0. 985	Ref.	1.000	0.998	0.999
	p-value	<0.001*	Ref.	<0.001*	<0.001*	<0.001*
MDRD1	Rho	0. 984	1.000	Ref.	0.999	0.999
	p-value	<0.001*	<0.001*	Ref.	<0.001*	<0.001*
CKD-EPI	Rho	0. 989	0.999	0.998	Ref.	0.999
	p-value	<0.001*	<0.001*	<0.001*	Ref.	<0.001*
CKD-EPI1	Rho	0. 988	0.999	0.999	0.999	Ref.
	p-value	<0.001*	<0.001*	<0.001*	<0.001*	Ref.

The regression analysis of demographic factors and selected cardiovascular risk factors associated with the various equations is presented in Table 10, including both unadjusted and multiple regression results. The study revealed that for the CG equation, age and albuminuria were the only significant factors associated with its predicted values. In contrast, the MDRD and MDRD1 equations were significantly associated solely with the presence of albuminuria. The CKD-EPI and CKD-EPI1 equations, however, were significantly associated with both age and albuminuria. These findings suggest that the factors influencing kidney function estimates differ between equations, with CG being affected by both age and albuminuria, while the MDRD equations are primarily associated with albuminuria, and the CKD-EPI equations are influenced by both demographic factors and albuminuria.

**Table 10:** Regression analysis of factors associated with CrCl, CG, MDRD, MDRD1, CKD-EPI, and CKD-EPI 1 *Significant Ref. = reference or baseline group, HTN = hypertension, BMI = body mass index, CG = Cockroft-Gault, MDRD= Modification of Diet in Renal Disease, MDRD1 = Modification of Diet in Renal Disease without race correction, CKD-EPI = Chronic Kidney Disease Epidemiology Collaboration, CKD-EPI1 = Chronic Kidney Disease Epidemiology Collaboration without race correction, CrCl = creatinine clearance

Variable	Unadjusted		Multiple regression	
	Coefficient (B)	P-value	Coefficient (B)	P-value
(Constant)	Ref.	Ref.	67.121	0.001
Age	-0.528	0.016*	-0. 585	0.010*
Sex	-3.837	0.574	5.599	0.449
Residence	11.514	0.529	10.980	0.531
BMI	0.115	0.863	0.447	0.518
HTN	-15.204	0.027*	-9.145	0.214
Diabetes	-6.964	0.847	-14.788	0.673
Connective tissue disease	24.318	0.501	13.156	0.706
Albumin	-0.008	0.002*	-0.009	0.001*
CG				
(Constant)	Ref.	Ref.	59.336	0.001
Age	-0.478	0.013*	-0.528	0.007*
Sex	-7.058	0. 238	2.225	0.727
Residence	11.636	0.469	12.046	0.465
BMI	0.451	0.439	0.655	0.272
HTN	-15.330	0.001*	24.306	0.419
Diabetes	-7.855	0.804	-13.543	0.654
Connective tissue disease	37.555	0.235	-9.610	0.131
Albumin	-0.007	0.001*	-0.007	0.001*
MDRD				
(Constant)	Ref.	Ref.	85.654	0.079
Age	-0.353	0.097	-0.381	0.074
Sex	-3.190	0.629	-5.804	0.415
Residence	12.773	0.470	-9.642	0.568
BMI	-0.197	0.759	-0.030	0.964
HTN	19.467	0.004	16.351	0.025*
Diabetes	-4.982	0.887	-21.187	0.513
Connective tissue disease	32.355	0.354	17.597	0.600
Albumin	-0.008	-0.001*	-0.008	0.001*
MDRD1				
(Constant)	Ref.	Ref.	58.869	0.081
Age	-0.288	0.101	-0.312	0.076
Sex	-2.528	0.643	-4.948	0.401
Residence	10.418	0.476	-7.806	0.576
BMI	-0.160	0.763	0.029	0.958
HTN	16.125	0.004	13.561	0.025*
Diabetes	-4.245	0.883	-17.789	0.524
Connective tissue disease	27.036	0.348	14.837	0.592
Albumin	-0.007	0.001*	-0.007	0.001*
CKD-EPI				
(Constant)	Ref.	Ref.	80.878	0.058
Age	-0.496	0.027*	-0.517	0.021*
Sex	-3.706	0.597	-5.974	0.423
Residence	14.154	0.451	-10. 813	0.540
BMI	-0.325	0.632	-0.012	0.986
HTN	21.964	0.002*	18.276	0.017*
Diabetes	-6.918	0.852	-22.844	0.518
Connective tissue disease	35.464	0.338	20.001	0.569
Albumin	-0.008	0.002*	-0.009	0.001*
CKD-EPI1				
(Constant)	Ref.	Ref.	89.909	0.058
Age	-0.428	0.027*	-0.446	0.021*
Sex	-3.157	0.602	-5.167	0.422
Residence	12.065	0.456	-9.187	0.547
BMI	-0.290	0.621	-0.022	0.971
HTN	18.879	0.002	15.683	0.018*
Diabetes	-6.427	0.841	-20.120	0.510
Connective tissue disease	30.909	0.333	17.684	0.560
Albumin	-0.007	0.002*	-0.008	0.001*

## Discussion

The GFR is widely acknowledged as the best index for renal function [12]. GFR estimating equations are vital tools in evaluating renal function, but these equations must be validated across different populations for their clinical relevance. Accurate GFR estimation is crucial for diagnosing and staging CKD, facilitating appropriate management and providing prognostication. Literature on the performance of GFR estimating equations in CKD patients is scarce in Northern Nigeria, and most available studies from other regions in the country have focused on population screening rather than assessing the equations' performance in CKD patients.

This study demonstrates that GFR predictive equations can be used to assess renal function in CKD patients, particularly when GFR is low. Additionally, it reveals that the latest equations may not always be the best, as the CG equation outperforms the CKD-EPI equation. Overall, the findings contribute to the body of knowledge regarding the validity of predictive equations for CKD patients and compare the performance of different equations within the same cohort.

Demographics

The mean age of participants was 51.1±15.5 years, consistent with the observed national average for CKD incidence in Nigeria. This contrasts with developed countries, where CKD predominantly affects individuals over 65 years. Several factors explain this difference, such as the genetic makeup of people of Black ancestry, which is associated with a higher CKD prevalence and poorer prognosis. Notable genetic variations include the APOL-1 gene polymorphism, which is linked to focal segmental glomerulosclerosis, HIV nephropathy, and diabetic nephropathy [23,24]. The high prevalence of sickle cell trait is also a risk factor for CKD, as it is associated with hematuria and abnormal tubular function. Additionally, the poor socio-economic status in developing countries contributes to infections and infestations, which are risk factors for CKD. Chronic glomerulonephritis is particularly common in this environment [4]. These factors may explain the relatively higher prevalence of CKD in younger populations in developing countries like Nigeria compared to developed countries. [25].

There was a slight preponderance of male participants in the study, which contrasts with findings from the United States Department of Health and Human Services [25] and a systematic review of CKD prevalence in Nigeria by Chukwuonye et al. [26], both of which report a higher CKD prevalence in women. The male-dominant finding in this study could be attributed to economic empowerment, which influences healthcare access. Moreover, male patients are more likely to progress to end-stage kidney disease, which may explain the higher number of males, as they tend to present at later disease stages.

The majority of participants were urban dwellers, reflecting the higher CKD prevalence in urban areas compared to rural ones. The hospital's urban location offers better access for city dwellers, and the study design also favored urban patients who were more willing to participate in 24-hour urine collection, unlike rural patients who often declined due to the distance.

The most common co-morbidity observed was hypertension, which is both a cause and consequence of CKD [26]. Obstructive uropathy, found in approximately 10% of male participants, is another notable co-morbidity, highlighting the high prevalence of prostatic enlargement in middle-aged men. A significant observation was the co-existence of diabetes mellitus in patients with clinical and imaging features indicative of hypertensive nephrosclerosis.

Clinical features

The relatively short duration of CKD in this study is consistent with the general observation that most CKD patients seek care late [27]. Additionally, a significant portion of participants had stage 5 CKD. Diabetic kidney disease was the most common cause of CKD, in line with global trends [3,28,29]. However, this differs from many studies in Nigeria, where chronic glomerulonephritis and hypertension are typically the leading causes of CKD [4,29,30]. This suggests that the epidemiology of CKD in Nigeria is changing, similar to other developing countries like India, where Westernized lifestyles and diets have led to an increased incidence of diabetes mellitus and, consequently, diabetic kidney disease [31]. Alebiosu et al. [4] noted a similar trend in 2006, indicating that diabetic nephropathy is becoming a significant cause of ESRD in Nigeria.

Less than a fifth of participants had a positive family history of CKD. The overall mean BMI was in the overweight range, with females having a significantly higher BMI than males (p<0.001). However, there was no significant association between BMI and CKD according to any of the estimation equations in regression analysis. The mean SBP and DBP were higher than the benchmark for diagnosing hypertension (140/90 mmHg), indicating the high prevalence of hypertension among participants. Elevated blood pressure was found to be associated with all predictive equations in regression analysis.

Laboratory features

Over three-fourths of participants had positive dipstick proteinuria, with 52% showing an ACR >300 mg/g, and up to 82% having ACR >30 mg/g. This high incidence suggests that the dipstick test has a high positive predictive value for proteinuria detection, with a performance rate of 75.7%. Although dipstick testing is prone to observer error and has a high false discovery rate, it remains a valuable initial screening tool in CKD assessment [32-37]. This finding aligns with Ulasi et al. [27] community-based study, which reported a proteinuria prevalence of 16.2% in CKD patients, with values increasing in later stages.

The median serum creatinine was significantly elevated above the normal reference range, as expected in a group of CKD patients, with a substantial portion in stage 5 disease. The median CrCl, corrected to 1.73 m² BSA, was used to categorize participants into different CKD stages. Over a quarter were in stage 5, which is consistent with the late presentation observed in other studies in Nigeria [27,29,30].

Glomerular filtration rate equations

The median estimated GFR derived from the CG, MDRD, and CKD-EPI equations closely matched the measured 24-hour CrCl. In contrast, the values obtained from MDRD1 and CKD-EPI1 were significantly lower than the measured 24-hour CrCl (p≤0.001 and p=0.020, respectively). These findings suggest that CG, MDRD, and CKD-EPI can be used with a reasonable degree of confidence in estimating GFR within the studied population.

When the results from the predictive equations were plotted against the measured 24-hour CrCl using Bland-Altman analysis, CG demonstrated the least bias and the highest precision (0.12±14.40 mL/min). The number of measurements outside the 95% limits of agreement ranged between 8 and 11, with CG having the fewest outliers (n=8). Discordances with measured CrCl occurred at GFR values >48 mL/min for CG and CKD-EPI, and >45 mL/min for MDRD, MDRD1, and CKD-EPI1. Although CG and CKD-EPI performed slightly better at detecting reduced renal function at higher GFRs, the clinical significance of this finding is minimal, as these values fall within the same CKD staging category.

These results align with previous studies. Agaba et al. [36] reported that CG had the least bias and best precision when compared to MDRD, with discordance thresholds at 30 mL/min and 40 mL/min, respectively. Similarly, Dimandopoulos et al. [38] observed that predictive equations performed best at GFR stages 4 and 5, with correlations diminishing at higher GFR values. CrCl correlated strongly with eGFR derived from CG, CKD-EPI, CKD-EPI1, and MDRD, with correlation coefficients of r=0.949, 0.939, 0.938, and 0.936, respectively. Despite the lack of statistically significant differences, CG emerged as the least biased equation for estimating CrCl. Similar observations were made by Abefe et al. [37] in Southwestern Nigeria, where CG showed a good correlation with measured CrCl, and MDRD did not demonstrate superiority over CG.

Significant negative correlations were found between the mean values of the predictive equations and the urinary ACR. This inverse relationship is expected, as albuminuria is a marker of CKD progression, with higher albumin excretion rates observed in advanced CKD stages. Studies in Nigeria have consistently demonstrated an association between low eGFR and increasing albuminuria, reinforcing the interplay between declining renal function and proteinuria [27,39,40].

There was a significant correlation between CG, MDRD, and CKD-EPI across CKD stages, except for stage 1, where MDRD1 significantly underestimated GFR. CG was the only equation that correlated with measured CrCl at stage 1, while all equations showed stronger correlations at stages 4 and 5, with increasing coefficients as GFR declined. This finding aligns with previous reviews by Coresh et al. [41,42], who reported that predictive equations become less accurate at GFR values above 60 mL/min, with bias increasing at higher GFRs.

Interestingly, while CKD-EPI has been shown to outperform MDRD at higher GFRs in other populations, the present study suggests that CG and CKD-EPI may be more appropriate for estimating renal function in CKD patients with reduced GFR. The discrepancy between studies by Eastwood et al. [20] and Shittu et al. [43], where MDRD and CKD-EPI overestimated GFR when adjusted for race, may be attributable to differences in population characteristics, as their cohorts included more individuals with normal renal function.

This study demonstrates strong agreement among predictive equations, particularly at GFR values <45 mL/min, with CG showing the least bias and best precision. While MDRD1 overestimated GFR, it had the highest number of measurements within 30% of the measured CrCl, supporting its accuracy in lower GFR ranges. The CG and CKD-EPI equations may be better suited for clinical use in this population, especially in patients with advanced CKD. Age and hypertension were significant factors influencing eGFR values, with CG showing greater underestimation in elderly patients. Despite subtle differences, all tested equations exhibited good predictive performance, reinforcing their utility in routine clinical practice. However, given the variability in equation performance across populations, local validation remains essential to ensure accurate GFR estimation and optimal CKD management.

This study is limited by the use of CrCl to eGFR rather than inulin clearance or radioisotope methods, which are considered the gold standard for measuring GFR. Although CrCl remains a widely used and practical alternative, its accuracy may be influenced by factors such as muscle mass, tubular secretion, and incomplete urine collection. Future studies may benefit from employing gold-standard techniques for more precise GFR assessment.

## Conclusions

This study confirms that GFR prediction equations, CG, MDRD, and CKD-EPI, provide reliable estimates of renal function in CKD patients, particularly at lower GFR stages. The CG equation showed the least bias and best precision among the equations analyzed. Albuminuria was significantly associated with CKD stage and GFR estimates. The findings suggest that these equations should be validated locally to ensure accurate CKD diagnosis and staging, which is crucial for optimal management. Further research is needed to validate these results in larger, population-based studies and explore the influence of genetic and socioeconomic factors on CKD progression and outcomes.

## Appendices

Appendix A

Consent Form

My name is Dr. Bakare Olawale, a resident doctor in the Department of Medicine at Jos University Teaching Hospital. I am conducting a study titled: “Evaluation of Glomerular Filtration Rate Prediction Equations in the Assessment of Renal Function in Chronic Kidney Disease in Nigeria.”

Study Purpose

This study aims to determine the most accurate equation for estimating glomerular filtration rate (GFR), a crucial measure of kidney function. The CKD-EPI equation, newly introduced, has not yet been validated in Jos, highlighting the need for this research.

Study Procedure

You will be asked to answer some questions in a questionnaire. Please provide accurate responses. Participation requires a one-day hospital admission (on a date convenient for you) to collect your urine over a 24-hour period. We will provide a collection container. You should discard the first urine at the start (0 hour) and collect all subsequent urine until the 24th hour the next day.

Approximately 5 mL of blood will be drawn around the time you complete your urine collection. This will be done with minimal discomfort and under the researcher’s supervision. Afterward, you will undergo a clinical examination, and both blood and urine samples will be analyzed. The results of this study will contribute to improving the accuracy of CKD diagnosis and assessing disease severity.

Potential Harm, Risks, or Discomfort

There are no significant risks associated with this study. However, you may experience slight discomfort or minimal pain during blood sample collection.

Right to Refusal or Withdrawal

Participation is voluntary. You have the right to withdraw from the study at any time, even after signing the consent form, without affecting the quality of care you receive at the hospital.

Confidentiality

All information provided will be kept strictly confidential. Your identity will not be disclosed, and any identifiable information will only be published with your explicit consent. Additionally, all costs related to the study, including admission and investigations, will be covered by the researcher at no expense to you.

Statement of Consent

I, (initials) ______________________, have asked questions about this research and received satisfactory answers. I choose to participate in this study.

I agree: {  }

I decline: {  }

Thank you.

Signature of Participant or Relation: _________________ Date: ________________

Signature of Witness: ____________________________ Date: ________________

Signature of Investigator: _________________________ Date: ________________

Researcher’s Phone Number: 08054246662

Ethics Committee Chairman’s Phone Number: 08037001392

Appendix B

Questionnaire

**Table 11:** The evaluation of glomerular filtration rate prediction equations in the assessment of renal function in chronic kidney disease in Nigeria

Date_________________		Serial number_________________	
Demographic Data			
Initials_________________	Hospital number_________________	Age (years)_________________	Date of birth_________________
Sex (✓): ☐ Male ☐ Female	Ethnicity	Occupation: ☐ Student ☐ Unemployed ☐ Trader ☐ Farmer ☐ Civil Servant ☐ Retired ☐ Others: ____________	
Educational Level: ☐ None ☐ Primary ☐ Secondary ☐ Tertiary ☐ Postgraduate ☐ Informal		Residence: ☐ Urban ☐ Suburban ☐ Rural	
Clinical History			
How long have you been diagnosed with CKD? _____________ years			
Family history of CKD: ☐ Yes ☐ No	Cause of CKD: ☐ CGN ☐ Hypertension ☐ Diabetes ☐ Connective Tissue Disease ☐ HIV ☐ Obstructive Uropathy ☐ Cystic Kidney Disease ☐ Others: _____________		
Medications: ☐ None Antihypertensives: ☐ ACEI/ARB ☐ CCB ☐ β-Blockers ☐ Diuretics ☐ Centrally Acting ☐ Vasodilators ☐ Others: _____________ Hematinics: ☐ Parenteral Iron ☐ Oral Iron ☐ EPO ☐ Folic Acid ☐ Others: _____________ Hypolipemics: ☐ Statins ☐ Fibrates ☐ Nicotinic Acid ☐ Cholestyramine ☐ Others: _____________ Hypoglycemic Agents: ☐ Insulin ☐ Biguanides ☐ Sulfonylureas ☐ SGLT2 Inhibitors ☐ DPP4 ☐ Others: _____________ HAART: ☐ Yes ☐ No If yes, what regimen? _____________ Others: ☐ Specify: _____________			
Co-morbidities			
Hypertension: ☐ Diabetes: ☐ Chronic Liver Disease: ☐ Obstructive Uropathy: ☐ Connective Tissue Disease: ☐ Others: ☐ Specify: _____________			
Physical Examination			
Weight: _____________ kg	Height: _____________ m	Body mass index (kg/m²): _____________	
Pulse rate: _____________ /min	Blood pressure: Systolic _____________ mmHg	Diastolic _____________ mmHg	
Laboratory Investigation			
Urine volume in 24 hours: _____________ mL	Urinary creatinine: _____________ µmol/L	Serum creatinine: _____________ µmol/L	
Urine albumin:			
Glucose ☐ Protein ☐ Blood ☐ Leukocytes ☐ Others: _____________ S.G.: _____________ pH: _____________	Creatinine clearance: _____________ Estimated GFR (Cockcroft-Gault): _____________ Estimated GFR (MDRD): _____________ Estimated GFR (MDRD1): _____________ Estimated GFR (CKD-EPI): _____________ Estimated GFR (CKD-EPI1): _____________ ACR: _____________		

Appendix C

Operational Definitions

Chronic kidney disease (CKD): Defined as a documented abnormality in kidney structure or function that persists for at least three months, evidenced by imaging, abnormal serum creatinine, or abnormal urinalysis. These were confirmed through medical records and participant questionnaire responses.

CKD is staged based on the measured 24-hour creatinine clearance (CrCl), which estimates the glomerular filtration rate (GFR). The staging is as follows: Stage 1 indicates a normal GFR of over 90 mL/min/1.73 m², while stage 2 indicates a mildly decreased GFR of 60-89 mL/min/1.73 m². Stages 3a and 3b indicate moderately decreased GFR, with values of 45-59 mL/min/1.73 m² and 30-44 mL/min/1.73 m², respectively. Stage 4 indicates a severely decreased GFR of 15-29 mL/min/1.73 m², and stage 5 indicates kidney failure, with a GFR of less than 15 mL/min/1.73 m².

Albuminuria: Defined as a positive dipstick protein and an albumin-creatinine ratio >30 mg/g.

Hypertension: Defined as systolic blood pressure (SBP) >140 mmHg or diastolic blood pressure (DBP) >90 mmHg.

Measured creatinine clearance: Defined as $UCr&times;VPCr\frac{UCr \times V}{PCr}PCrUCr&times;V​$, corrected to 1.73 m² BSA for each participant.

Cockcroft-Gault (CG) equation: Defined as creatinine clearance derived from the electronic version of the CG equation, corrected to 1.73 m² BSA for each participant.

Statistical Definitions

Bias: The mean difference between estimated GFR (using each equation) and measured 24-hour creatinine clearance.

Precision: The standard deviation of the difference between the mean estimated GFR and measured 24-hour creatinine clearance.

Accuracy: The percentage of measurements within 30% of the limits of the measured 24-hour creatinine clearance using each equation.
